# Multiplexed bead-based assay for the simultaneous quantification of human serum IgG antibodies to tetanus, diphtheria, pertussis toxin, filamentous hemagglutinin, and pertactin

**DOI:** 10.3389/fimmu.2023.1190404

**Published:** 2023-06-05

**Authors:** Vishal Rathod, Laxmikant Kadam, Manish Gautam, Prabhu Dasu Gumma, Kevin Marke, Cathy Asokanathan, Alex Douglas-Bardsley, Laura Hassell, Sachin Bhandare, Sumit Gupta, Sameer Parekh, Pramod Pujari, Harish Rao, Hitt Sharma, Umesh Shaligram, Sunil Gairola

**Affiliations:** ^1^ Clinical Bioanalytical Laboratory, Serum Institute of India Pvt. Ltd., Pune, Maharashtra, India; ^2^ Science, Research and Innovation, Medicines, and Healthcare Products Regulatory Agency, South Mimms, United Kingdom

**Keywords:** diphtheria, enzyme-linked immunosorbent assay (ELISA), Luminex (xMAP) method, multiplex immunoassay (MIA), pertussis, tetanus

## Abstract

**Background:**

Luminex bead-based assays offer multiplexing to test antibodies against multiple antigens simultaneously; however, this requires validation using internationally certified reference standards. Therefore, there is an urgent need to characterize existing reference standards for the standardization of multiplex immunoassays (MIAs). Here, we report the development and validation of an MIA for the simultaneous estimation of levels of human serum immunoglobulin G (IgG) antibodies for pertussis toxin (PT), filamentous hemagglutinin (FHA), pertactin (PRN), diphtheria toxoid (DT), and tetanus toxoid (TT).

**Methods:**

The MIA was assessed using a panel of human serum samples and WHO reference standards. The WHO reference standards were also studied for suitability in the MIA. Purified antigens (PT, FHA, PRN, DT, and TT) were coupled to the spectrally unique magnetic carboxylated microspheres. The method was validated in accordance with the United States Food and Drug Administration (US FDA), European Medicines Agency (EMA), and the International Committee of Harmonization Multidisciplinary (ICH M10) guidelines, and parameters such as precision, accuracy, dilutional linearity, assay range, robustness, and stability were assessed. Method agreements with commercially available IgG enzyme-linked immunosorbent assay (ELISA) assays were also evaluated. In addition, the study assessed the level of correlation between the IgG levels estimated by the MIA and the cell-based neutralizing antibody assays for PT and DT.

**Results:**

We identified that an equimix of WHO international standards (i.e., 06/142, 10/262, and TE-3) afforded the best dynamic range for all the antigens in the MIA. For all five antigens, we observed that the back-fitted recoveries using the four-parameter logistic (4-PL) regression fits ranged between 80% and 120% for all calibration levels, and the percentage coefficient of variation (% CV) was < 20%. In addition, the difference in mean fluorescence intensity (MFI) between the monoplex and multiplex format was < 10% for each antigen, indicating no crosstalk among the beads. The MIA also showed good agreement with conventional and commercially available assays, and a positive correlation (> 0.75) with toxin neutralization assays for PT and DT was observed.

**Conclusion:**

The MIA that was calibrated in accordance with WHO reference standards demonstrated increased sensitivity, reproducibility, and high throughput capabilities, allowing for the design of robust studies that evaluate both natural and vaccine-induced immunity.

## 1 Introduction

Despite good immunization coverage, sporadic cases of vaccine-preventable diseases such as whooping cough/pertussis (1), diphtheria (2), and tetanus (3) are reported globally. Novel combination vaccines for children and adult populations targeting pertussis (subunit/acellular or whole-cell based), diphtheria, and tetanus antigens continue to be developed and tested. Serology continues to provide valuable immunogenicity and diagnostic data on pertussis-based combination vaccines. Levels of antigen-specific immunoglobulin G (IgG), as quantified by validated enzyme-linked immunosorbent assay (ELISA) methods, serve as a correlate of protection for acellular pertussis (aP)-based combination vaccines (4). Immunogenicity testing of aP-based vaccines has been historically carried out using commercially available diagnostic kits. Although validated using international standards (5), the commercial kits have concerns about lot-to-lot variability due to sourcing of required materials and the quality of coating antigens used. In addition, running single-antigen ELISA kits are time and labor-intensive and require large quantities of sera, which are often challenging to procure (6–10).

Multiplex immunoassays (MIA) represent an alternative approach for quantifying IgGs in a highly sensitive, specific, and reproducible manner. Several studies have reported the usefulness of multiplex platforms for immunogenicity assessment of aP-based combination vaccines (11–15). A study evaluating a tetraplex microsphere assay for pertussis antigens showed high concordance with an in-house ELISA (16). The assay demonstrated that the MIA could measure pertussis antigens quickly and accurately (16). However, few studies are available wherein aP antigens are multiplexed with diphtheria and tetanus antigens. A previous study has reported on a pentaplex Luminex assay covering aP, diphtheria, and tetanus antigens to evaluate the immunogenicity of combination vaccines in mouse models (13). The multiplex assay offers the advantage of lesser turnaround time in simultaneously detecting several antigens utilizing lesser sample volumes. The assay is also accurate, has a high-throughput, and reduces material costs and labor compared with conventional ELISA (17).

Microsphere-based Luminex immunoassays use spectrally distinct fluorescent microspheres as the solid support matrix (18). The target antigens are coupled onto the support matrix to simultaneously measure antibodies against multiple analytes from a single reaction well, thus reducing the analysis time, cost, and sample volume (18). The MIAs for human vaccines must be developed and validated to report results in units traceable to an appropriate international reference standard (19). Notably, for aP combination vaccines, three different WHO international reference standards from the National Institute for Biological Standards and Control (NIBSC), namely, 06/142 [pertussis toxin (PT), filamentous hemagglutinin (FHA), and pertactin (PRN)], 10/262 (diphtheria), and TE-3 (tetanus) are recommended for the calibration of immune assays for the determination of levels of antibodies against pertussis, diphtheria, and tetanus antigens, respectively. Although these international standards are suited for monoplex assays (20, 21), MIAs require a single standard to provide unitage to all the targeted antigens. Therefore, we have characterized the existing international standards, namely 06/142, 10/262, and TE-3, according to their suitability in the MIA. Such characterization will provide opportunities for using these reference standards in MIAs and support standardizing and pooling clinical results across multiple studies with greater confidence and reproducibility.

Our study reports on the development and validation of a pentaplex magnetic bead-based Luminex assay for evaluating antibody IgG concentrations against PT, FHA, PRN, diphtheria toxoid (DT), and tetanus toxoid (TT) in human serum samples using international reference standards. Method validation was designed as per the United States Food and Drug Administration (US FDA) (22), European Medicines Agency (EMA) (23), and International Council of Harmonization Multidisciplinary (ICH M10) (24) guidelines. Method agreement with commercially available assays was also evaluated. In addition, the study analyzed the correlation of the MIA with toxin-neutralization functional antibody assays for diphtheria and pertussis toxin. Ours is the first study to report the characterization of existing international standards (ISs) for MIAs. The unitages established for ISs will also be helpful for the development of MIAs on other platforms.

## 2 Materials and equipment

### 2.1 Antigens and reagents

Purified PT, FHA, PRN antigens, DT, and TT were sourced from the Serum Institute of India Pvt. Ltd. (SIIPL, India). All antigens were tested for content and purity. The protein content of the antigens was estimated using a validated bicinchoninic acid (BCA) assay (25). Purity was tested using a validated sodium dodecyl-sulfate polyacrylamide gel electrophoresis (SDS-PAGE) assay. In accordance with the manufacturer’s recommendation, antigens were stored in aliquots at temperatures of –20°C or lower. R-phycoerythrin (R-PE) conjugated to anti-human antibody was obtained from Southern Biotech, United States of America (USA). Beads (carboxylated microspheres) were procured from Luminex Corporation, USA, and 1-ethyl-3-(3-dimethyl aminopropyl) carbodiimide (EDAC) was obtained from Bio-Rad Laboratories, India. Sulfo-N Hydroxysulfosuccinimide (sulfo-NHS) was procured from ThermoFisher Scientific, USA, and bovine serum albumin (BSA) was obtained from Sigma Aldrich, India. Tween-20 was purchased from SD Fine Chem Limited, India.

### 2.2 International standards and reference reagents

WHO ISs and reference reagents were purchased from the NIBSC, UK. Four WHO reference standards were used in the study: 06/142, 10/262, TE-3, and 13/240. The unitages of the reference standards are reported in the international unit (IU)/mL, traceable to the international reference standard. The WHO reference reagent for pertussis antiserum human (06/142) is a freeze-dried preparation of pooled human serum with an assigned anti-PT IgG content of 106 IU/ampoule, an anti-FHA IgG content of 122 IU/ampoule, and an anti-69 K IgG content of 39 IU/ampoule. The WHO IS for diphtheria antitoxin human (10/262) is a freeze-dried preparation of normal human IgG with a diphtheria antitoxin potency of two IU/ampoule. For tetanus, the first WHO International Standard for Anti-Tetanus Immunoglobulin Human (TE-3) is a freeze-dried preparation of human tetanus immunoglobulin with an assigned unitage of 120 IU/ampoule (26). TE-3 has been replaced by a second WHO international standard, 13/240: a freeze-dried preparation with an assigned unitage of 45 IU/mL. These reference standards were supplied in ampoules and were reconstituted as per manufacturer recommendations. The unitages in IU/mL for these standards as per the certificate of analysis are summarized in **Table 1**.

**Table 1:** WHO reference standards with respective unitages.

Standards	Batch No.	Antibodies (IgG)	IU/ml
**Pertussis Antiserum**	**06/142**	PT	106
		FHA	122
		Anti-69K (Pertactin)	39
**Diphtheria Antitoxin**	**10/262**	DT	2
**First IS - tetanus immunoglobin**	**TE-3**	TT	120
**Second IS - tetanus immunoglobulin**	**13/240**	TT	45

Anti-69kDa, pertactin; DT, diphtheria toxoid; FHA, filamentous hemagglutinin; IgG, immunoglobulin G; IS, international standard; IU/mL, international units per milliliter; NIBSC, National Institute for Biological Standards and Control; PT, pertussis toxin; TT, tetanus toxoid; WHO, World Health Organization.

The values in bold are used to indicate the codes for the WHO NIBSC reference standards.

### 2.3 Reference standard development for the pentaplex assay

**Figure 1:**
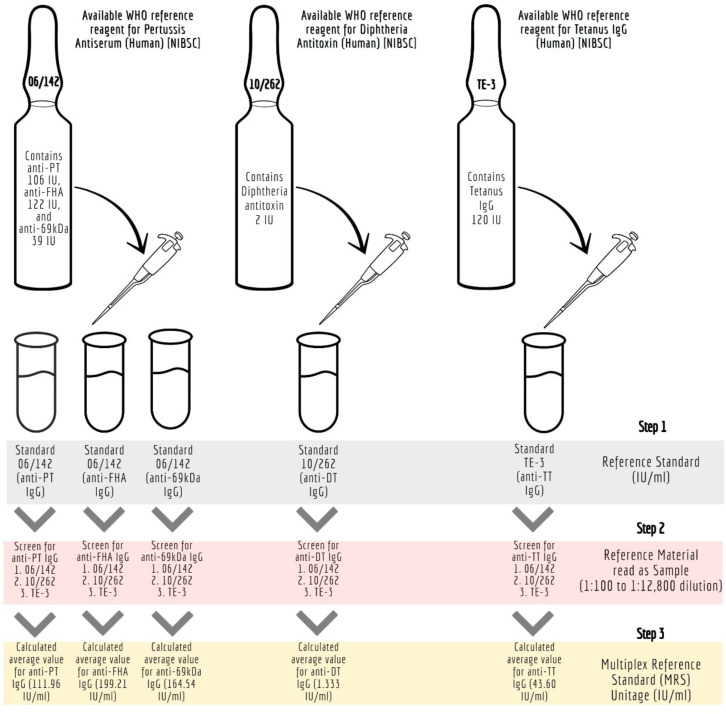
Development of Multiplex Immunoassay (MIA) reference standard (MRS) using international reference standards. anti-69kDa, pertactin, DT, diphtheria toxoid; FHA, filamentous hemagglutinin; IU/ml, international units per millilitre; mIU/ ml; milli-international units per millilitre; MRS, Multiplex Reference Standard; NIBSC, National Institute for Biological Standards and Control; Anti-69K, Pertactin; PT, pertussis toxin; TT, tetanus toxoid.

The details of the ISs (WHO reference standards) used in the study are provided in **Table 1**. These reference standards are human serum preparations (high titers) from vaccinated healthy volunteers, and they are assigned IUs based on multiple global inter-laboratory studies. These standards are developed to calibrate immunoassays focused on determining antibodies against the target antigens. The MIA requires a reference standard that could provide unitage against all five antigens. Therefore, all the reference standards were evaluated to assess their suitability for use in the pentaplex assay. We used the three WHO international reference reagents, that is 06/142, 10/262, and TE-3, to prepare the multiplex reference standard (MRS) by mixing equal proportions of them (1:1:1). Reference standard development for MIA followed WHO recommendations on developing secondary reference standards (27). Briefly, 06/142, 10/262, and TE-3 were screened for antibodies against all five antigens. The antibody levels against PT, FHA, and PRN in each of these standards were quantified using 06/142 as a reference standard. The antibodies against TT and DT in these standards were quantified using the TE-3 and 10/262 reference standards, respectively. Six runs were carried out to quantify IgG levels against TT, DT, PT, FHA, and PRN in these reference standards. The final content (average of six runs) of each reference standard was used to calculate the final stock concentration of MRS. **Figure 1** provides the schematic presentation of the approach used to develop MRS using international reference standards.

### 2.4 Internal quality controls

Internal quality controls (IQCs) stock standards were prepared by mixing equal volumes (1:1) of 10/262 and TE-3. The stock standard unitages were determined against the MRS as PT (110.43 IU/mL), FHA (228.70 IU/mL), PRN (212.06 IU/mL), DT (1.737 IU/mL), and TT (61.08 IU/mL). Using the stock standard, five different IQC levels (IQC-1 to IQC-5) were prepared using Luminex assay buffer. Acceptance limits were established by repeated testing of the IQCs (*n* = 15). Acceptable ranges for the estimates were set as the mean ± 2 standard deviations (SD) of the IgG concentrations of each antigen.

### 2.5 Human serum samples for method validation

Serum samples (unvaccinated and vaccinated) used for method validation were collected from healthy volunteers aged > 18 years working at SIIPL, India, after obtaining informed consent. The selected sera (*n* = 15) samples for the study are presented in **Table 2**. The selected sera samples were of various concentrations, that is negative, low, medium, and high concentrations. All serum samples were used in accordance with local regulations and guidelines and approved by the Independent Research Ethics Committee, Pune (IEC No. IRECP/015/2020). The sera samples were tested using an MIA to quantify the levels of antibodies against all five antigens. Based on concentrations, eight different panels were designed for method validation. Panel 1: Samples for precision and accuracy containing high, medium, and low levels of IgG; Panels 2–4: Samples for selectivity containing negative or low levels of IgG; Panel 5: Samples for dilution linearity containing high levels of IgG; Panel 6: Samples for stability at 2–8°C and room temperature from precision and accuracy panel; Panel 7: Samples for freeze-thaw stability (S1-S8: Reference standard) from precision and accuracy panel; Panel 8: Samples for solution stability, *samples from the precision and accuracy panel.

**Table 2:** Sera panel used for assay validation.

Sample no.	Panel 1	Panel 2	Panel 3	Panel 4	Panel 5	Panel 6	Panel 7	Panel 8
	(Vaccinated Sera)	(Non-vaccinated Sera)	(Hemolytic and Lipemic Sera)	(Antibody Depleted Human sera)	(High Titre Sera)	(Vaccinated sera for Stability)	(Vaccinated sera and MRS)	(MRS and IQC)
**1.**	DU0001/B/2020/IA	AM0015/NB/2020/IA	Hemolytic sera	Blank Human Sera	DU0001/B/2020/IA*	DU0001/B/2020/IA*	S1	S1
**2.**	RS0002/B/2020/IA	DG0016/NB/2020/IA	Lipemic sera		RS0002/B/2020/IA*	RS0002/B/2020/IA*	S2	S2
**3.**	AS0004/B/2020/IA	ST0017/NB/2020/IA			AS0004/B/2020/IA*	SS0007/B/2020/IA*	S3	S3
**4.**	AN0005/B/2020/IA	SS0018/NB/2020/IA			AN0005/B/2020/IA*	AS0009/B/2020/IA*	S4	S4
**5.**	SP0006/B/2020/IA	PO0019/NB/2020/IA			MRS	RK0010/B/2020/IA*	S5	S5
**6.**	SS0007/B/2020/IA	SH0020/NB/2020/IA				IQC 1	S6	S6
**7.**	AZ0008/B/2020/IA					IQC 2	S7	S7
**8.**	AS0009/B/2020/IA					IQC 3	S8	S8
**9.**	RK0010/B/2020/IA						DU0001/B/2020/IA*	IQC 1
**10.**	IQC 1						RS0002/B/2020/IA*	IQC 1
**11.**	IQC 2						SS0007/B/2020/IA*	IQC 1
**12.**	IQC 3							IQC 2
**13.**	IQC 4							IQC 2
**14.**	IQC 5							IQC 3
**15.**								IQC 3

Panel 1 contains samples for precision and accuracy containing high, mid, and low levels of IgG. Panels 2–4 contain samples for selectivity containing negative or low levels of IgG. Panel 5 contains samples for dilution linearity containing a high level of IgG. Panel 6 contains samples for stability at 2–8°C and RT from precision and accuracy. Panel 7 contains samples for freeze—thaw stability (S1–S8: Reference standard) from precision and accuracy. Panel 8 contains samples for solution stability, *samples from the precision and accuracy panel. IQC, internal quality control; MRS, multiplex reference standard.

## 3 Methods

### 3.1 Assay development

#### 3.1.1 Coupling of antigens to carboxylated microspheres

The antigens (PT, FHA, PRN, DT, and TT) were coupled to the spectrally unique magnetic carboxylated microspheres using established and commercially available coupling procedures. Two commercially available procedures were evaluated: the first was based on the Luminex cookbook published previously by Kadam L et al. (13, 28) and the second was based on the commercially available kit from AnteoTech (Australia) (29). For the coupling of antigens, microspheres were activated with a carbodiimide derivative, EDAC hydrochloride-containing buffered solution. The intermediate carboxyl groups that formed on the beads as a result of this reaction with EDAC were stabilized using a sulfo-NHS solution. This was followed by three washing steps using a magnetic separator. The respective antigens were added to the activated beads and kept in the dark for 2 h under constant mixing (15–30 rpm). The resulting mixture was washed, and the supernatant was discarded during every washing step. After three stages of pelleting and washing, coupled beads were blocked using 1% BSA in 1x phosphate-buffered saline (PBS) buffer for 30 min and kept in a storage buffer (0.1% w/v BSA in PBS containing 0.05% sodium azide and 0.02% Tween 20).

**Figure 2:**
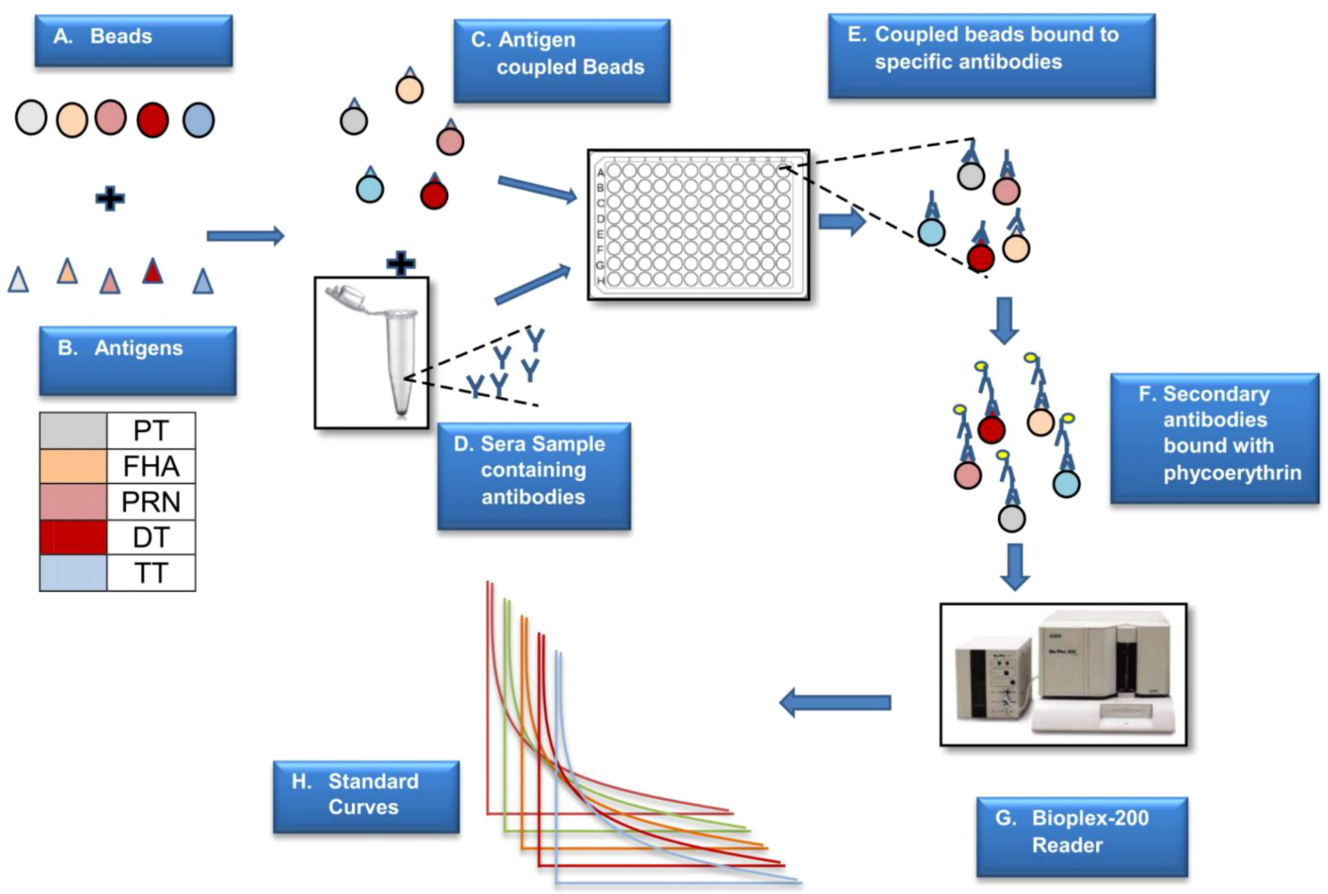
Schematic presentation of MIA. The above figure is a step-by-step representation of MIA (5-plex) wherein different beads are used to couple the antigens **(A)**. Antigen coupled beads are incubated with the sera sample to capture specific antibodies **(B–E)**. This binding is monitored by positive reaction with PE labelled secondaryantibody **(F)**. The reaction on beads is analysed using a specific reader **(G)** wherein the reported fluorescence is directly proportional to the amount of antigen specific antibody in the sera sample **(H)**. The assay is based on external reference standard and quantification is performed using standard curve fitted using logistic curve **(H)**. Different colors are used to depict five different antigens.

The coupling, activation, and storage buffers used were procured from AnteoTech, Australia. The beads were activated using the activation buffer for 60 min. The antigens to be coupled were prepared using a conjugation buffer. The antigens were mixed with the activated beads and incubated at room temperature for 60 min. The unbound antigens were removed by washing them thrice with the wash buffer. Beads were then incubated in a blocking buffer (0.1% BSA in conjugation buffer) for 60 min, then stored in a storage buffer. **Figure 2** presents the schematic details of MIA.

#### 3.1.2 Characterization of WHO reference standards for the development of MRS

Monoplex bead-based assays were carried out for unitage assessment of 06/142, 10/262, and TE-3, as detailed in **Figure 1**. Monoplex assay here refers to a setup wherein only one target antigen is added instead of five different beads. The design of the monoplex assay is identical to MIA, with the difference that the assay uses monovalent beads (~ 4,000 beads per well were used). Briefly, two-fold serial dilutions (from 1:1,000 to 1:1,28,000) of the respective human reference standards (WHO reference standards 06/142, 10/262, and TE-3) were performed eight times and were added to the monovalent beads. Test serum samples (WHO reference standards) were also assessed at multiple serial dilutions starting from 1:1,000 to 1:1,28,000. Assay blanks were included in the plate as a control. All incubation conditions, numbers of washes, buffers, and instrument settings used were the same as those used for the pentaplex assay (**Section 3.1.5**).

#### 3.1.3 Verification of assigned unitages using commercially available assays

Commercially available Conformité Européenne (CE)-certified ELISA assay kits (IBL, USA, and Euroimmun, Germany) were used to confirm the unitages assigned to all the antigens in MRS. ELISA assays were performed for the PT, FHA, PRN, DT, and TT antigens. The IBL ELISA kits (PRN, DT, and TT) contained the calibrators and positive and negative controls. The WHO standards 06/142, 10/262, and TE-3 were used as test serum samples. The assays were performed as per the manufacturer’s instructions. The samples were diluted from 1:100 to 1:12,800, added to the pre-coated plate, and incubated for 1 h at 37°C. Following this, 100 μL of enzyme conjugate (peroxidase-labeled anti-human IgG) was added to each microplate well. This mixture was incubated for 30 min at room temperature and washed thereafter. A volume of 100 μL of chromogen/substrate solution was added to each of the microplate wells. This was incubated for 15 min at room temperature, following which 100 μL of stop solution was added. The optical density (OD) was read at 450 nm using the Biotek ELISA reader (USA). The OD values within the linear part of the curve were converted to IU/mL by interpolation from a four-parameter logistic (4-PL) standard curve.

The Euroimmun (PT, FHA) ELISA test kit was used for the *in vitro* quantification of human antibodies of the IgG class in serum. In the first reaction step, diluted samples were incubated in the wells, and positive samples contained specific IgG antibodies bound to the antigens. A second incubation was carried out using an enzyme-labeled anti-human IgG (enzyme conjugate), catalyzing a color reaction to detect the bound antibodies. Photometric measurement of the color intensity was conducted at a wavelength of 450 nm and a reference wavelength between 620 nm and 650 nm and read within 30 min of the stop solution being added. The results of this assay were compared with those of the bead-based assay.

#### 3.1.4 Unitage confirmation at the National Institute for Biological Standards and Control laboratory

For characterization, MRS was also tested at the National Institute for Biological Standards and Control (NIBSC) laboratory using conventional ELISA assays for PT, FHA, PRN, DT, and TT. For PT, FHA, and PRN, after each step, plates were washed with PBS (pH 7.4) containing 0.05% v/v Tween 20 (phosphate-buffered saline solution with Tween 20, PBST), and all incubations, unless otherwise specified, were carried out at room temperature. Briefly, 96-well ELISA plates (Nunc MaxiSorp, Thermo Fisher Scientific, USA) were coated with 100 µL of two µg/mL solution of either PT (NIBSC in-house), FHA (NIBSC JNIH-4) or PRN (NIBSC 18/154) in carbonate buffer (pH 9.5 containing 0.035 M sodium hydrogen carbonate, 0.015 M sodium bicarbonate, and 7.4 mM sodium azide) per well overnight. Plates were blocked with 100 µL of PBST containing 10% fetal bovine serum (FBS) for 1 h, followed by incubation with samples and reference (WHO reference reagent 06/142) at a starting dilution of 1:100 in blocking buffer for 1.5 h. Two-fold serial dilutions were performed using a blocking buffer as the diluent. Following this, antigen-specific IgG antibodies were detected with 100 µL of rabbit anti-human IgG labeled with horseradish peroxidase (Sigma, A-8792), diluted at 1:2,000 in a blocking buffer for 1.5 h. Finally, 100 µL of 1% 3,3′,5,5′-tetramethyl-benzidine (TMB) substrate (Sigma, T-2885) in dimethyl sulfoxide (DMSO) and 0.03% hydrogen peroxide (Sigma, H1009) in acetate buffer (pH 6.0) was added and color was developed for 15 min, after which 50 µL 1M sulfuric acid was added to stop the reaction. The OD was measured at 450 nm using a Multiskan ELISA plate reader (Molecular Devices, UK). Antibody responses for the MRS were calculated relative to the WHO reference material by parallel line analysis (log OD vs. log dose), using a minimum of three sequential points from the linear section of the dose–response curves and expressed in IU/mL.

For DT and TT, ELISA plates were coated overnight at 4°C with either 100 µL per well of DT (NIBSC 13/212, 3.7 flocculation units/mL) or 100 µL per well of TT (NIBSC 02/126, 0.5 flocculation units/mL) diluted in carbonate buffer (0.05 M, pH 9.6). The ELISA plates were washed three times with PBST and blocked with 150 µL of PBST containing 5% (w/v) dried skimmed milk powder (PBSTM-5%) for 1 h at 37°C. Following a second wash in PBST, serial two-fold dilutions of the WHO reference material (10/262 for diphtheria ELISA and TE-3 for tetanus ELISA) and MRS in PBSTM-1% were prepared in the plate (final volume 100 µL), and the plates were incubated at 37°C for 2 h. Plates were washed as described previously, and antigen-specific IgG antibodies were detected using a horseradish peroxidase-conjugated rabbit anti-human IgG antibody (Sigma, A-8792) diluted 1:2,000 in PBSTM-1%. After incubation for a further 1 h at 37°C and a final wash, 100 µL per well of substrate solution containing 0.5 mg/mL 2,2′-azino-bis (3-ethylbenzthiazoline-6-sulfonic acid) diammonium salt (ABTS, Sigma A9941) and 0.008% hydrogen peroxide (Merck, 107209) in 0.05 M citric acid buffer (pH 4.0) was added, and this mixture was allowed to develop for up to 30 min. The OD was measured at 405 nm using a Multiskan ELISA plate reader (Molecular Devices, UK). Antibody responses for the MRS were calculated relative to the WHO reference material. Analysis of variance was used to determine if there was any significant deviation from the linearity or parallelism of the dose–response relationship (*p* < 0.01).

The unitages were compared against the acceptance criteria of assigned unitages of MRS within a 30% variability margin that was attributed to the use of different assay platforms, antigens, and inter-laboratory variations.

#### 3.1.5 Pentaplex immunoassay

The MRS (mix of 06/142, 10/262, and TE-3) was prepared as detailed in Section 2.3 and was used as an assay calibrator. The MRS was serially diluted two-fold from 1:333 to 1:42,624. The test sera samples were diluted serially two-fold from 1:100 to 1:12,800 using Luminex assay buffer and tested at multiple dilutions. The filter plate was used for the assay. The multivalent beads were added in each well at 50 μL/well (~ 4,000 beads per well) and aspirated. From the dilution plate, 50 μL of reference standard and samples were transferred in duplicate to the filter plate, incubated in the dark for 60 min at 37°C, and shaken at 150 rpm. The plate was aspirated and washed thrice with 100 μL assay buffer. To each well, 50 μL of a 1:100 diluted R-PE goat anti-human antibody was added, incubated in the dark for 30 min at 37°C, and shaken at 150 rpm. The plate was aspirated and washed thrice with 100 μL Luminex assay buffer, and the microspheres were resuspended in 100 μL assay buffer. The assay blank and IQCs were run in each plate. The plate was read in the Protein Suspension Array System (Bioplex-200). The reference standard’s backfit of 70%–130%, percentage coefficient of variation (% CV) of ≤ 20%, and IQCs acceptance criteria were used as system suitability criteria.

#### 3.1.6 Toxin neutralization assays

A toxin neutralization assay (*n* = 3) was performed to verify the ability of antibodies in the serum samples to neutralize active pertussis and diphtheria toxins. The Chinese hamster ovary (CHO) cell-clustering assay based on the induction of clusters in non-confluent CHO cell cultures by aP toxin was performed to assess toxin neutralization. Serially diluted sera samples were incubated with a known concentration of toxins at 37°C for 60 min. After incubation, CHO cells with a concentration of 3 × 10^4^ cells/mL were added to all wells of the antigen–antibody mixture, and the plates were incubated at 37 ± 1°C for approximately 48 h. Following this, CHO cells were observed for clustering under an inverted microscope. The highest dilution of sera, which showed cluster neutralization, was recorded as the sample titer. A positive score was assigned when 10 or more CHO cell clusters were evident within a single well (30).

The Vero cell assay has been used to determine the protective level of diphtheria antitoxin in human sera (31). The metabolic activity and survival of Vero cells in cultures are inhibited by diphtheria toxin, and diphtheria antitoxins may neutralize this effect of the toxin in serum samples. Titration of serum samples on Vero cells in the presence of fixed amounts of diphtheria toxin was carried out in three independent assays (*n* = 3). Reading of the Vero assay was based on a microscopic examination of cells to determine the color change in the wells of microtiter plates from red to yellow due to the metabolic formation of acid. To prepare diphtheria toxin, serum dilutions were prepared in a microtiter plate in minimum essential medium (MEM) supplemented with 10% FBS. Diphtheria toxin at a lethal tissue culture (LTC) dose of 100 was added and incubated for 45 min to 1 h at room temperature (20–25°C) for toxin neutralization. We prepared a Vero cell suspension containing 3.5–4.5 × 10^5^ cells/mL, added 100 µL to the 96-well microtiter plates, incubated for 5 days at 36 ± 1°C under 5% CO_2_ atmosphere, and then observed the cells to determine if metabolic inhibition caused by a non-neutralized toxin had occurred. A Vero cell assay was performed to determine the neutralization of diphtheria toxin. Fixed sera dilutions of diphtheria toxin were prepared and the titer of the serum sample was calculated by comparing the test results with standard diphtheria antiserum. A factor of the highest dilution, showing metabolic inhibition, was multiplied by 0.2 (limit of detection of this method) to report the results in IU/mL.

### 3.2 Assay validation

The assay was validated based on the FDA, EMA, and ICH M10 guidelines for bioanalytical methods.

#### 3.2.1 Assay specificity

Assay specificity was evaluated in three different runs by (a) inhibition experiments and (b) comparing the MFI difference between the multiplex assay (five antigens simultaneously) and monoplex assay (each antigen individually). For inhibition experiments, MRS was incubated independently with each purified antigen (PT, FHA, PRN, DT, and TT) and with a mixture of all five antigens (PT + FHA + PRN + DT + TT) for 1 h before analysis. The percentage reductions in MFIs due to purified antigens (specific antigens) and a mix of antigens were compared to determine assay specificity.

#### 3.2.2 Assay selectivity

The method’s selectivity was evaluated in three independent runs using three human serum matrices: (i) matrix 1—non-vaccinated sera (panel 2; samples 1–6), (ii) matrix 2—hemolytic and lipemic matrix (panel 3; samples 1–2), and (iii) matrix 3—antibody-depleted human sera (panel 4; sample 1) as mentioned in **Table 2**. These matrices are representative of negative or low-concentration sera. Matrices 1 and 2 were spiked with different concentrations of reference standard and tested at concentrations of 1:400 (high), 1:6,400 (medium), and 1:12,800 (low). Matrix 3 was spiked with the MRS and IQC. Recovery of spiked samples from the different matrices was calculated with the acceptance criteria within the range of 70%–130% of expected concentrations.

#### 3.2.3 Precision

The assay precision was evaluated over 3 days and six runs for different analysts, days, and lots of coupled beads and phycoerythrin (PE) (**Table 2**, Panel 1). Intra-assay precision refers to the variability observed for the same day. Inter-assay precision refers to the variability in experiments performed on different days by different analysts using different lots of beads and PE lots. The assay precision was reported in terms of the % CV.

#### 3.2.4 Accuracy

Accuracy was assessed over 3 days and six runs using a panel of sera samples (**Table 2**, Panel 1). These samples were tested at different concentrations in six assays spread over 3 days using three different bead lots and read by two analysts. The estimates were compared with the assigned unitages to determine the accuracy. The resulting IgG concentration of each serum sample was calculated and compared with the assigned values, with an acceptance criterion of recovery of between 70% and 130%.

#### 3.2.5 Dilution linearity

Dilution linearity was evaluated in three different runs using panel 5 (**Table 2**). Assay dilutability was assessed in three independent runs, using two-fold dilutions starting from 1:100 until the serum sample was quantifiable. Recovery was calculated as a percentage difference between the observed and assigned concentrations. Linearity was considered acceptable if said dilution complied with an acceptable % CV of duplicates (i.e., < 20%) and if the dilution-corrected concentrations were within 70%–130% of the assigned values.

#### 3.2.6 Assay range

The reference standard for the determination of the assay range was evaluated in six runs by two-fold serial dilutions of the MRS from 1:333 to 1:42,624. The assay range for each antigen was determined using estimates from precision, accuracy, and dilution linearity, after which the most stringent lower and upper concentration limits complying with acceptable accuracy (70%–130%) and precision (< 20% CV) and dilutional accuracies of between 70% and 130% were selected. The assay range was also supported by back-calculated concentrations of calibration standards. The back-calculated concentrations were to be within 70%–130%.

#### 3.2.7 Limit of detection and limit of quantification

The limit of detection (LOD) and limit of quantification (LOQ) for each of the five antigens were determined in three runs using curve-fitted MFI at the minimum detectable response (i.e., a three-fold increase in the minimum baseline response) and minimum quantifiable response (i.e., a five-fold increase in the minimum baseline response).

#### 3.2.8 Robustness

Robustness data on IQCs concerning incubation time, temperature, bead lot, and PE lots were assessed. Five IQCs were used to analyze robustness and results were used to estimate the % CV for each parameter tested. The % CV of observed vs. estimated concentrations concerning deliberate parameter variations was assessed. The following parameters were studied during the robustness assessment: assay step 1 and step 2 incubation times, temperature, different lots of secondary antibodies (PE lots), and beads. Both step 1 (incubation with beads; 50–70 min) and step 2 (incubation with PE; 20 to 40 min) incubation time assays and primary and secondary incubation temperatures (32–42°C) were evaluated. Two different PE lots and bead lots were also evaluated for robustness.

#### 3.2.9 Stability study

The stability of the serum samples, MRS, and IQCs from Panel 6 was monitored at 25°C and 2–8°C. Serum samples were assessed for stability at 25°C for up to 72 h and at 2–8°C for up to 168 h. Freeze–thaw stability was evaluated at –20°C for serum samples and MRS (**Table 2**, panel 7). The samples were aliquoted and exposed to freeze–thaw cycles wherein the sera samples were thawed for 2 h by placing the samples at room temperature (i.e., no higher than 25°C). Later, sera samples were frozen for 24 h at –20°C before thawing. The percentage differences between assigned and observed concentrations were determined for the stability study. The impact of freeze–thaw cycles was evaluated with an acceptance criterion of ± 30% difference relative to the assigned concentrations.

#### 3.2.10 Solution stability

The reference standards and the IQCs from panel 8 (**Table 2**) were used to evaluate the solution stability. The solution stability of the assay was determined by analyzing the assay plates at pre-determined intervals of 0, 6, 8, 12, and 24 h. The results obtained at different intervals were compared with the precision study set to determine the hold time of the plate with the acceptance criteria of ≤ 20%.

#### 3.2.11 Edge effect

The edge effect was evaluated in three runs using the assay control. IQC 3 was placed in each well of the 96-well filter plate. The % CV of MFIs was calculated for the 96-well plate, with an acceptance criterion of % CV ≤ 20 for all antigens.

#### 3.2.12 Statistical analysis

A log/log-linear regression model was used to fit the reference standard curve. Calibration curves were generated using the 4-PL logistic fit; the values for back-fitted recoveries were set between 70% and 30%, and the % CV values were set at ≤ 20%. At least 75% of the calibration standards, or a minimum of six standards, had to meet these criteria. Statistical analyses were performed using Microsoft Office Excel 2019 and statistical software GraphPad Prism 7.05. The results generated by MIA were compared with sera neutralization assays using linear regression analysis in Microsoft Office Excel 2019.

The following formula was used for method validation parameters:

The percentage recovery for selectivity assessment was calculated as follows:


$$\begin{array}{c} \left(observed\;concentration\;of\;spike\;sample-observed\;concentration\;of\;unspiked\;sample\right)\\ ÷expected\;concentration\times 100 \end{array}$$

The following equations were used to calculate MFI for the determination of LOD and LOQ:


$$\begin{array}{c} Minimum\;\mathrm{detectable}\;response=average\;blank\;human\;serum\;MFI+\\ \left(3\times SD\;of\;blank\;serum\;MFI\right) \end{array}$$

Minimum quantifiable response=5×(MFI at minimum detectable response)

The lowest quantifiable response was multiplied by 200 (minimum two sera dilutions, i.e., 100 and 200) to obtain the LOQ in IU/mL.

## 4 Results

### 4.1 Assay development

#### 4.1.1 Optimization of bead coupling procedures

**Figure 3:**
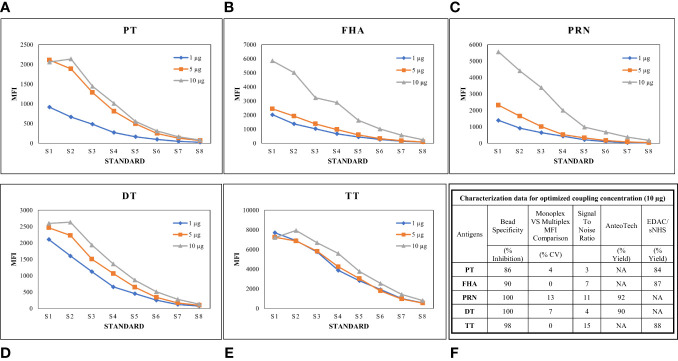
Optimization of Bead coupling procedures. **(A–E)** represents dilution vs MFI graphs at three different beads coupling concentrations for PT, FHA, PRN, DT and TT antigens. 10ug/ml concentration was found optimum for all the antigens. **(F)** represents the characterization data of bead coupling at 10ug/ml with respect to suitability parameters of bead specificity, signal to noise ratios, yields and MFI comparisons. DT, Diphtheria Toxoid; FHA, Filamentous haemagglutinin; MFI, Mean fluorescence intensity; PRN, Pertactin; PT, Pertussis Toxin; TT, Tetanus Toxoid; S17ndash;S8, Standards.

Luminex-based MIA involves using beads (microspheres) with different fluorochromes detected in unique wavelength regions using a particular instrument known as Luminex-200. Targeted proteins are coupled onto these beads following a specific conjugation procedure. We previously reported the optimized conditions for connecting these antigens, wherein two coupling methods were optimized, namely, EDAC/Sulfo-NHS using the Luminex cookbook, and the AMG kit from AnteoTech. A similar procedure was used in this study (13). A coating concentration of 10 µg/mL for PT, FHA, PRN, DT, and TT antigens was used in the study. In this study, coupling procedures were further evaluated concerning performance in the human serum matrix. Suitability was assessed, wherein for each bead set, MRS (positive for all five antigens) was used to generate a standard curve (eight serial dilutions; two-fold) and at each point, the MFI was assessed to demonstrate the linearity across titrations. The coating concentration of 10 µg/mL was found to be suitable, as a good dynamic range of 1:100 to 1:42,624 of MRS was observed for all five antigens. We also evaluated the incubation time (beads with sera) for all five antigens. It was noted that an incubation time of 1 h was suitable, ensuring that a good signal-to-noise ratio was achieved for all five antigens. Luminex assay buffer was optimized to work with a minimum sera dilution of 1:100. The use of Luminex assay buffer with a composition of 1% BSA, 0.2% sodium azide, 0.1% Tween 20, and PBS was conducive to optimal assay performance. The optimized coating concentrations were further evaluated for possible interference by comparing the MFIs of mono and multiplex conditions using human sera. MFIs were comparable: a ≤ 20% difference in % CV was observed, supporting their suitability for use in MIA. Bead cross-reactivity was also assessed using inhibition experiments, wherein the percentage reductions in MFIs were noted against individual antigens as compared with positive uninhibited control. All beads achieved a homologous inhibition of over 85%, which further indicates the suitability of the coupling procedures used in the assay (**Figure 3**).

#### 4.1.2 Characterization of WHO reference standards for the development of MRS

MRS represents an equimolar mixture of three WHO reference standards. WHO reference standards are serum preparations (high titers) that are sourced from vaccinated healthy volunteers. The unitages of these reference standards are assigned based on multiple global inter-laboratory studies and are more suited to calibrating single antigen immunoassays. An MIA being carried out in a single well requires a reference standard that could provide unitages against all five antigens. Previous studies have used in-house reference standards using sera samples from clinical studies. This study reports on the development of MRS using international standards, as the sourcing of clinical samples in sufficient quantities may not be feasible for all laboratories. The development of MRS was carried out using the approach outlined in **Figure 1**. The approach comprises three major steps. The first step is the screening study. The objective of the screening study is to evaluate proposed reference standards for the presence of antibodies against other antigens. This is important as these reference standards are sourced from vaccinated volunteers and most vaccines are combination vaccines. Therefore, even though the reference standard provides unitages for a specific antigen, the sera may also be positive for other antigens in the panel. For example, TE-3 provides unitages for tetanus antibodies; however, the sera were also positive for antibodies against PT, FHA, PRN, and diphtheria. This is expected as vaccines containing tetanus antigens are combination vaccines that also include pertussis and diphtheria antigens. **Table 3** provides the results of the screening study for all the WHO reference standards. These results will be important for all the laboratories working on multiplex immunoassays. In the second step, based on the results of the screening study, MRS was established and unitages were assigned. The unitages assigned to the MRS using data from six independent runs are provided in **Table 4**. In the third step, the assigned unitages to international reference standards and the MRS for all five antigens were also verified using commercially available ELISA assays. These commercially available assays report the unitages traceable to the specific international reference standard. **Table 5** provides the comparative assessment of unitages assigned by bead-based assay and commercially available methods. The unitages by bead-based assay were in good agreement with the commercial ELISA assays, as the variabilities of all unitages were ≤ 20%. In addition, the MRS was also sent to NIBSC for characterization studies, and the results of these indicated that there was excellent agreement (**Table 6**) between the NIBSC estimates and the multiplex assay estimates, as all the estimates were within the acceptable % CV range of ≤ 20%. NIBSC laboratories used the conventional monoplex plate-based ELISA method to confirm the unitages provided by MIA, which further supports the concordance of the MIA with conventional monoplex ELISA assays.

**Table 3:** Characterization of WHO reference standards for development of multiplex reference standard.

WHO reference Standard	IU/ml				
	PT	FHA	PRN	DT	TT
**06/142**	**100.70 (95%)**	**116.99 (96%)**	**36.75 (94%)**	0.18	2.16
**10/262**	70.57	117.22	155.02	**1.94 (97%)**	11.47
**TE-3**	164.61	363.42	301.85	1.88	**117.18 (98%)**
**13/240**	153.25	262.64	202.13	6.55	**40.55 (90%)**

WHO reference standards were screened for IgG antibodies against PT, FHA, PRN DT, and TT using a bead-based assay. Values in bold indicate the observed concentrations, and values in parenthesis indicate their percentage agreement with the official unitages. DT, diphtheria toxoid, FHA, filamentous hemagglutinin; IU/mL, international units per milliliter; PRN, pertactin, PT, pertussis toxin; TT, tetanus toxoid; WHO, World Health Organization.

**Table 4:** Assigned unitages of multiplex reference standard.

Antigen	Assigned unitage (IU/ml)	%CV
**PT**	111.96	**14**
**FHA**	199.21	**15**
**PRN**	164.54	**18**
**DT**	1.333	**9**
**TT**	43.60	**3**

Multiplex reference standard is an equimolar mixture of TE-3, 10/262, and 06/142. Based on the screening study, unitages were assigned to MRS. Values are a representation of mean IU/mL (N = 6 assays). Values in bold indicate the % CV of six assays. % CV, percentage coefficient of variation; DT, diphtheria toxoid, FHA, filamentous hemagglutinin; IU/mL, international units per milliliter; MRS, multiplex reference standard; PRN, pertactin, PT, pertussis toxin; TT, tetanus toxoid.

**Table 5:** Characterization of WHO reference standards using commercially available ELISA kits and percent agreement with MIA.

Samples	PT			FHA			PRN			DT			TT		
	Bead Based Assay	ELISA	% Agreement	Bead Based Assay	ELISA	% Agreement	Bead Based Assay	ELISA	% Agreement	Bead Based Assay	ELISA	% Agreement	Bead Based Assay	ELISA	% Agreement
06/142	101	115	**88**	117	108	**108**	37	43	**85**	0.2	0.2	**89**	2	2	**108**
10/262	71	61	**116**	117	106	**111**	155	146	**106**	1.9	1.7	**114**	11	14	**82**
TE-3	165	151	**109**	363	317	**115**	302	260	**116**	1.7	1.4	**135**	117	147	**80**
13/240	153	145	**106**	263	272	**97**	202	191	**106**	6.6	5.9	**112**	41	44	**93**

The bead-based assay showed excellent agreement with the commercially available assay. Values in bold indicate the percentage agreement between bead-based monoplex assays and commercially available ELISA assays. DT, diphtheria toxoid; ELISA, enzyme-linked immunosorbent assay; FHA, filamentous hemagglutinin; MIA, multiplex immunoassay; MRS, multiplex reference standard; PRN, pertactin; PT, pertussis toxin; TT, tetanus toxoid.

**Table 6:** Verification of assigned unitages of MRS using commercial, NIBSC, and multiplex assays.

Antigen	SIIPL Bead-Based Assay (IU/ml)	NIBSC ELISA Assay (IU/ml)	Commercial ELISA kit (IU/ml)	%CV
**PT**	112	122	134	**9**
**FHA**	199	169	201	**9**
**PRN**	165	119	174	**19**
**DT**	1.33	1.30	1.19	**6**
**TT**	44	43	47	**5**

%CV, percentage coefficient of variation; DT, diphtheria toxoid; ELISA, enzyme-linked immunosorbent assay; FHA, filamentous hemagglutinin; IU/ml, international units per millilitre; NIBSC, National Institute for Biological Standards and Control; PRN, pertactin; PT, pertussis toxin; SIIPL, Serum Institute of India, Pvt, Ltd; TT, tetanus toxoid; Values in bold indicates the %CV of three assays (Bead-based, commercial and NIBSC).

#### 4.1.3 Reference standard curve for MIA

**Figure 4:**
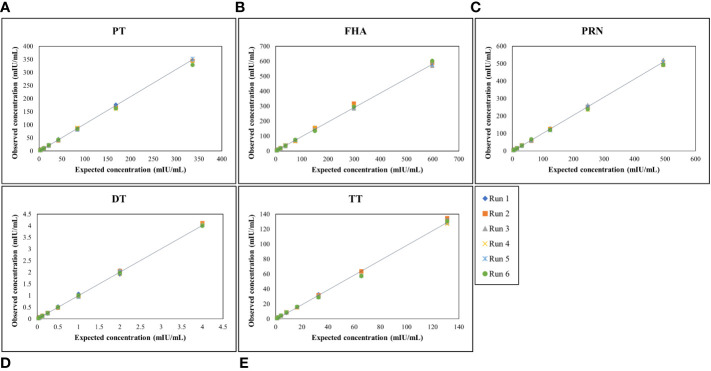
Dynamic range of MRS for each antigen. **(A–E)** represents the assay range of MRS for five antigens. The X-axis represents expected concentration (mIU/mL) whereas Y-axis represents obtained concentration (mIU/mL.). Data is representative of 6 runs. DT, Diphtheria Toxoid; FHA, Filamentous haemagglutinin; PRN, Pertactin; PT, Pertussis Toxin; TT, Tetanus Toxoid.

MRS serum with assigned IgG antibody concentrations for all five antigens, as indicated in **Table 4**, was used for the optimization of the reference standard curve. In any MIA, the reference standard curve should be optimized to cover a broad concentration range for all the antigens included in the MIA. Overall, eight separate two-fold dilutions of the MRS were performed and were fitted using a 4-PL fit. **Figure 4** shows the reference standard serum dilution profiles for each of the five antigens. Linearity of response was demonstrated using back-fitted recoveries, and all five antigens showed 80%–120% recoveries for all calibration levels. MRS covered a maximum possible concentration range of 2.63–336 mIU/mL for PT, 4.67–598 mIU/mL for FHA, 3.86–494 mIU/mL for PRN, 0.03–4 mIU/mL for DT, and 1.02–131mIU/mL for TT (**Table 7**). The lower limit (LL) and upper limit (UL) of the assay range were determined using estimates from accuracy, precision, and dilution linearity analysis (**Table 7**).

**Table 7:** Final assay range with a lower and upper limit of quantification.

Antigen	Precision (mIU/ml)		Accuracy (mIU/ml)		Dilutional linearity of sample (mIU/ml)		Dilutional linearity of standard (mIU/ml)		Calibration Curve range (mIU/ml)	
	Lower limit	Upper limit	Lower limit	Upper Limit	Lower limit	Upper limit	Lower limit	Upper limit	Lower Limit	Upper limit
**PT**	6.9	221	6.9	221	**2.63**	336	0.16	336	2.63	336
**FHA**	14.3	457	14.3	457	**4.67**	598	0.29	598	4.67	598
**PRN**	13.3	424	13.3	424	**3.86**	494	0.24	494	3.86	494
**DT**	0.11	3.4	0.11	3.4	**0.03**	4	0.003	4	0.03	4
**TT**	3.8	121.7	3.8	121.7	**1.02**	131	0.06	131	1.02	131

DT, diphtheria toxoid; FHA, filamentous hemagglutinin; PRN, pertactin; PT, pertussis toxin; TT, tetanus toxoid; mIU/ml, Milli-international unit per milliliter.

Data is representative of estimates in precision, accuracy, and dilution linearity validation parameters. Precision, accuracy, and dilution linearity estimates to support calibration curve range. Values highlighted in bold were used to estimate the lower limit.

### 4.2 Assay validation

The MIA was validated for specificity, selectivity, precision, accuracy, dilutability, LOQ, and stability using sera samples from vaccinated volunteers. The validation study design was based on the FDA, EMA, and ICH M10 guidelines for bioanalytical methods.

#### 4.2.1 Assay specificity

Specificity was demonstrated by (a) inhibition experiments and (b) comparing the MFI difference between the monoplex and multiplex assays. The MFI response (percentage difference) in monoplex and multiplex format was observed at < 10% for all five antigens (**Table 8**), which showed that there was no cross-reactivity between the beads. For inhibition experiments, the percentage inhibition of MFI of a positive serum sample following the addition of either an individual antigen or a mixture of antigens was assessed for all five antigens. The concentration of antigens used for the inhibition experiments was 2.62 µg for all five antigens. The addition of homologous antigens, either individually or in a mixture, resulted in an > 85% inhibition of signal for PT, PRN, FHA, DT, and TT antibodies (**Table 8**), indicating the high specificity of the assay in capturing the respective antibodies in the serum sample.

**Table 8:** Specificity of assay.

Antigen	MFI difference monoplex vs. multiplex	Inhibition	
	Difference (%)	Monoplex Inhibition (%)	Multiplex Inhibition (%)
**PT**	1	88	87
**FHA**	1	91	90
**PRN**	8	99	100
**DT**	8	99	100
**TT**	-1	99	99

DT, diphtheria toxoid; FHA, filamentous hemagglutinin; MFI, mean fluorescence intensity; PRN, pertactin; PT, pertussis toxin; TT, tetanus toxoid.

#### 4.2.2 Assay selectivity

The selectivity of the method was evaluated with respect to the use of different serum matrices for hemolytic, lipemic, non-vaccinated, and antibody-depleted sera. The assay high selectivity, as excellent spike recoveries (80%–120%) were observed in all the matrices (**Table 9**). No interference was observed in the assay for hemolytic and lipemic matrices covering up to 2.02 g/dL of hemoglobin and 275 mg/dL of total cholesterol, respectively.

**Table 9:** Selectivity assessment in different matrices (Panel 2-4).

Panel No.	Samples	Reference Standard Spike Level	Spike % Recovery				
			PT	FHA	PRN	DT	TT
**Panel 2**	Sample 1	High	97	101	98	97	101
		Middle	95	103	95	95	91
		Low	95	122	98	103	96
	Sample 2	High	92	93	91	91	95
		Middle	89	88	104	92	82
		Low	93	92	113	100	91
	Sample 3	High	98	102	97	97	104
		Middle	97	97	96	108	101
		Low	94	97	93	120	108
	Sample 4	High	92	95	91	91	95
		Middle	100	89	98	95	94
		Low	108	92	107	112	102
	Sample 5	High	105	107	99	101	105
		Middle	85	95	90	100	94
		Low	91	112	97	113	97
	Sample 6	High	100	106	103	100	110
		Middle	96	92	105	108	73
		Low	105	106	118	127	92
**Panel 3**	Sample 1	High	97	96	93	95	103
		Middle	94	95	92	97	108
		Low	97	100	95	108	125
	Sample 2	High	99	101	94	96	102
		Middle	102	104	98	107	113
		Low	100	114	104	125	131
**Panel 4**	Sample 1	IQC 1	111	107	104	104	92
		IQC 2	107	99	87	96	83
		IQC 3	100	105	97	97	83

DT, diphtheria toxoid, FHA, filamentous hemagglutinin; IQC, internal quality control; Panel 1, Panel for precision and accuracy; Panel 2, Panel for selectivity parameters; Panel 3, Hemolytic lipemic sera; Panel 4, Blank human sera; PRN, pertactin; PT, pertussis toxin; TT, tetanus toxoid.

Selectivity was assessed using spike recovery experiments in different serum matrices.

#### 4.2.3 Precision

Precision analysis suggested that the assay was precise for different analysts on different days using different lots of beads and PE. The % CV for the combined precision of the two analysts was below 20% for all five antigens (**Table 10**). Based on the data, the precision-based LLs and ULs ranged from 6.9 to 221 mIU/mL for PT, 14.3 to 457 mIU/mL for FHA, 13.3 to 424 mIU/mL for PRN, 0.11 to 3.4 mIU/mL for DT, and 3.8 to 121.7 mIU/mL for TT (**Table 7**).

**Table 10:** Precision and accuracy estimates.

Precision	*Analyst (% CV)					**Days (% CV)					***Bead Lot (% CV)				
	PT	FHA	PRN	DT	TT	PT	FHA	PRN	DT	TT	PT	FHA	PRN	DT	TT
**Sample 1**	12	10	11	12	10	10	10	13	12	11	9	10	13	16	8
**Sample 2**	8	8	9	5	7	8	7	11	11	11	5	5	8	11	8
**Sample 3**	11	10	11	11	8	16	14	17	15	18	14	12	7	13	18
**Sample 4**	7	9	8	5	13	15	10	12	13	18	9	8	15	13	15
**Sample 5**	12	11	9	7	3	11	8	16	11	12	6	3	6	14	8
**Sample 6**	7	10	9	6	1	12	9	17	13	13	7	11	11	12	8
**Sample 7**	5	8	9	8	8	12	13	11	10	11	10	10	10	9	9
**Sample 8**	7	9	10	6	14	12	15	12	11	14	7	15	4	5	6
**Sample 9**	4	5	5	7	13	11	10	10	10	11	8	7	7	6	6
**Sample 10**	7	6	7	7	10	11	6	8	7	14	9	4	6	7	19
**Sample 11**	6	10	6	7	6	10	11	8	8	9	9	12	7	7	7
**Sample 12**	7	8	7	7	8	10	10	9	8	8	8	8	7	6	6
**Sample 13**	10	10	8	10	8	11	11	10	10	9	10	8	8	8	7
**Sample 14**	11	13	11	15	13	11	14	12	14	12	8	11	9	15	9
Accuracy	*Analyst (% Recovery)					**Days (% Recovery)					***Bead Lot (% Recovery)				
**Sample 1**	96	102	106	98	104	91	98	99	96	100	92	96	97	99	102
**Sample 2**	94	97	111	91	102	91	91	102	90	95	91	88	96	94	95
**Sample 3**	107	100	101	103	118	105	97	89	98	110	99	89	84	92	100
**Sample 4**	86	92	91	90	112	89	90	87	96	101	90	90	85	95	95
**Sample 5**	99	94	106	96	108	96	94	100	104	106	95	93	92	109	107
**Sample 6**	104	109	106	110	112	100	106	97	106	104	99	103	88	96	99
**Sample 7**	93	99	101	101	106	92	94	96	99	102	92	93	90	96	103
**Sample 8**	100	106	104	111	110	90	95	95	102	99	83	90	90	96	95
**Sample 9**	99	104	92	92	97	90	94	87	86	93	85	92	86	84	92
**Sample 10**	92	106	93	98	93	101	110	96	95	96	100	111	93	94	93
**Sample 11**	90	106	93	100	83	96	109	96	96	88	97	108	95	97	87
**Sample 12**	91	95	95	101	86	98	96	97	98	92	98	99	98	101	90
**Sample 13**	89	91	94	104	94	96	94	96	98	93	93	93	96	100	88
**Sample 14**	90	94	94	102	97	95	98	100	98	94	100	100	100	100	100

Precision and Accuracy results are determined concerning different analysts, days, and bead lots. DT, diphtheria toxoid; FHA, filamentous hemagglutinin; PRN, pertactin; PT, pertussis toxin; TT, tetanus toxoid. Precision is determined in terms of % CV. Accuracy is reported in terms of % recovery. *Combined precision (% CV) and accuracy (% recovery) of analysts’ 1 and 2, **Combined precision and accuracy of 6 runs over 3 days, ***Combined precision and accuracy of multiple bead lots.

#### 4.2.4 Accuracy

Acceptable recoveries were observed within the range of 80%–120% for PT, FHA, PRN, DT, and TT antigens (**Table 10**). The accuracy-based LLs and ULs ranged from 6.9–221 mIU/mL for PT, 14.3–457 mIU/mL for FHA, 13.3–424 mIU/mL for PRN, 0.11–3.4 mIU/mL for DT, and 3.8–121.7 mIU/mL for TT (**Table 7**).

#### 4.2.5 Dilution linearity

**Figure 5:**
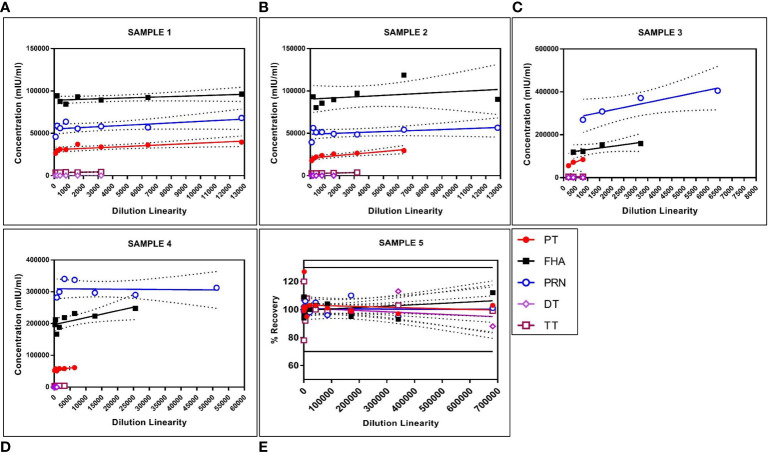
Dilution linearity of assay in high tire sera samples and MRS for PT, FHA, PRN, DT, and TT antigens. The X-axis represents the sample's dilutions, and the Y-axis represents the concentration observed in (mIU/ml). **(A–D)** represent dilution linearity graphs for high titer samples. **(E)** represents dilution linearity data for MRS. Sera samples and MRS shows no loss of dilution integrity over the dilution range. The dotted line in the figure represents the 95% confidence interval. DT, Diphtheria Toxoid; FHA, Filamentous Hemaagglutinin; MRS, Multiplex Reference Standard; PRN, Pertactin; PT, Pertussis Toxin; TT, Tetanus Toxoid.

The panel samples were tested in three independent runs across a series of sera samples ranging from a dilution of 1:100 to 1:681,984. No loss in dilution integrity was observed, with a two-fold increase in the dilution range recorded for all antigens (**Figure 5**).

#### 4.2.6 Assay range

The assay range was selected based on the estimates from precision, accuracy, and dilutional linearity study sets. The LL and UL of the assay range were established as ranging from 2.63 to 336 mIU/mL for PT, 4.67 to 598 mIU/mL for FHA, 3.86 to 494 mIU/mL for PRN, 0.03 to 4 mIU/mL for DT, and 1.02 to 131 mIU/mL for TT. The LL of an assay range was the lowest concentration that showed acceptable precision, accuracy, and dilution linearity in the experiments (**Table 7**).

#### 4.2.7 Robustness

The robustness of the assay was studied using IQCs covering the entire assay range. The critical assay parameters studied included incubation time with beads, incubation time with PE, incubation temperature of beads and PE, different lots of PE, and different bead lots. The % CV of observed versus expected concentrations was calculated for each IQC. The results demonstrated that concentrations of IQCs generated from the assays with deliberate variations were within the acceptable range of < 20% variability for all the antigens (**Table 11**).

**Table 11:** Assay robustness.

% CV											
Antigen	IQC	Step 1 Incubation Time		Step 2 Incubation Time		Incubation Temperature		Different PE Lots		Different Bead Lots	Edge Effect
		50 min	70 min	20 min	40 min	32°C	42°C	PE Lot 1	PE Lot 2	Bead Lot	
**PT**	**IQC 1**	6	10	4	3	2	5	3	1	9	NA
	**IQC 2**	9	7	2	6	3	9	7	4	9	NA
	**IQC 3**	6	8	5	6	1	1	4	0	8	5
	**IQC 4**	7	5	5	9	2	8	3	3	10	NA
	**IQC 5**	7	11	3	0	3	9	3	0	8	NA
**FHA**	**IQC 1**	6	2	4	7	13	8	9	10	4	NA
	**IQC 2**	0	12	3	5	4	1	8	3	12	NA
	**IQC 3**	1	16	2	4	4	1	1	8	8	5
	**IQC 4**	1	6	5	2	11	8	2	2	8	NA
	**IQC 5**	3	5	5	3	0	2	2	5	11	NA
**PRN**	**IQC 1**	6	9	4	5	0	3	3	4	6	NA
	**IQC 2**	8	6	6	6	2	9	8	8	7	NA
	**IQC 3**	7	8	6	5	2	1	9	5	7	4
	**IQC 4**	14	10	4	1	1	6	4	3	8	NA
	**IQC 5**	10	8	5	2	5	9	7	4	9	NA
**DT**	**IQC 1**	4	8	2	7	6	0	2	9	7	NA
	**IQC 2**	4	5	6	6	5	3	7	10	7	NA
	**IQC 3**	5	5	4	8	7	2	7	10	6	5
	**IQC 4**	7	4	3	0	2	3	5	12	8	NA
	**IQC 5**	9	12	2	0	4	4	6	10	15	NA
**TT**	**IQC 1**	13	25	15	19	17	9	19	16	19	NA
	**IQC 2**	6	6	11	16	13	17	15	11	7	NA
	**IQC 3**	10	14	4	12	1	12	3	3	6	2
	**IQC 4**	16	13	9	0	6	6	6	9	7	NA
	**IQC 5**	13	20	6	6	0	11	0	13	9	NA

Table reports % CV observed for deliberate variations in critical assay parameters for all the five antigens. % CV represents the percent difference between assigned and values observed post deliberate variation in parameter. Edge effect was studied with the IQC-3, being representative of concentration in mid region of the assay range.

DT, diphtheria toxoid; FHA, filamentous hemagglutinin; IQC, internal quality control; NA, not applicable; PE, phycoerythrin; PRN, pertactin; PT, pertussis toxin; TT, tetanus toxoid.

#### 4.2.8 Stability studies

Stability studies included assessment at different conditions including room temperature, 2–8°C and freeze–thaw (–20°C). The IQC and sera samples were found to be stable for up to 72 h and 168 h at room temperature and 2–8°C, respectively. In the freeze–thaw study, sera samples were found to be stable for up to 20 freeze–thaw cycles (**Supplementary material**, **Figure S1**, **Figure S2**).

#### 4.2.9 Solution stability

The results of analyzing assay plates at predetermined intervals of 0, 6, 8, 12, and 24 h suggest that a plate hold time of under 12 h would be suitable, as we observed an impact on PRN antigens after 12 h of plate hold time (**Supplementary material, Figure S3**).

#### 4.2.10 Edge effect

No variability was noted in the wells and all the MFIs were within the acceptable variability of 10% CV **(** **Table 11** **)**.

#### 4.2.11 Correlation with sera neutralization assays

**Figure 6:**
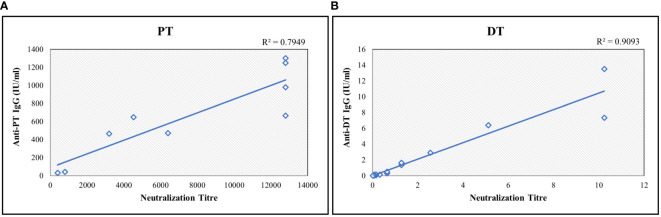
Correlation study between bead-based multiplex assay and toxin-neutralization assay for **(A)** PT and **(B)** DT. The neutralization titre is defined as highest serum dilution which shows the inhibition activity. Neutralization assay readout is end-point dilution wherein the highest dilution of sera sample showing the inhibition is reported. Each sample was tested 3 times. Correlation coefficient was determined using regression analysis. R^2^ values were determined for agreement between assays. DT, Diphtheria Toxoid; PT, Pertussis Toxin.

PT and DT are major virulence factors of *Bordetella pertussis and Corynebacterium diphtheriae*, respectively. Toxin neutralization assays using CHO and Vero cells for PT and DT were used to measure neutralization antibodies. These *in vitro* cell-based assays measured the functional antibodies. Ten serum samples were analyzed for correlations of results obtained with the MIA. Estimates exhibited positive correlations among the assays with correlation coefficients above 0.75 for both antigens (**Figure 6**).

## 5 Discussion and conclusion

The assessment of serum IgG responses to the antigens present in the aP-based combination vaccines has been reported mainly using conventional ELISA tests or commercial kits (7, 32). Such ELISA methods are expensive, time-consuming, and, most importantly, require considerable volumes of sera (33). Our study demonstrates an MIA for multiple applications, including serosurveillance and monitoring of vaccine immune responses. MIAs such as Luminex x-MAP^®^ and Meso Scale Diagnostics offer opportunities by providing rapid procedures for the simultaneous quantification of antibodies to multiple antigens with high sensitivity and selectivity using minimal amounts of sera samples. Luminex technology is based on the use of beads that facilitate the measurement of various analytes from a single sample (34). The beads are color-coded microspheres that contain different proportions of red and infrared fluorophores. These beads, when activated at a specific light spectrum, aid in the quantification of the analyte. Luminex technology allows for the use of both non-magnetic and magnetic beads. The use of magnetic beads in the assay was shown to have a high coupling efficiency and higher reproducibility due to lower inter-assay variation (35). In our study, we used magnetic beads for coupling the antigens and observed high coupling yields and minimum interferences from the matrices. The reproducibility of the coupling method is an essential factor for ensuring the consistency of test results, especially those from larger clinical trials. Ruling out the impact of the conjugation method on antigen epitopes is one of the prerequisites for developing bead-based immunoassays. A study by van Gageldonk PG et al. (11) described using commonly used conjugation protocols for PT, FHA, PRN, DT, and TT antigens. The two commercially available conjugation procedures evaluated in our study (Luminex cookbook and AnteoTech kit) for coupling the antigens to the beads demonstrated assay specificity and linearity for all antigens. The specificity experiments involving inhibition assays using homologous antigen confirmed that antigenic epitopes were unaffected by the coupling process, as the addition of 2.62 µg/mL of antigen inhibited signaling by > 85%. The robustness of the conjugation process was further demonstrated using three different lots of coupled bead assays, which demonstrated good reproducibility. It has previously been reported that MIAs’ improved performance and sensitivity are attributed to the control over the purity of antigens in the assays and the correlation of Luminex technology to single antigen ELISA using purified antigens (20). It was also noted that the purity of PT, FHA, and PRN antigens was critical to the assay. The in-house manufactured antigens with a purity of > 95% showed excellent results in the MIA. With tight control on the purity of target antigens and the use of magnetic beads, both coupling methods showed good agreement and were found to be suitable for the assay.

With the advent of MIA technologies and increasing regulatory expectations for validating clinical immunogenicity assays, multiplex assays must be validated against a recognized standard to provide uniformity and reproducibility. The NIBSC provided three reference standards, 06/142, 10/262, and TE-3, which had the unitages for aP, DT, and TT antigens. MIAs being carried out in a single well will require a reference standard that provides the unitages of all five antigens. As part of assay development, an equimolar mix of WHO reference standards was assessed as a possible reference standard for the multiplex assay. The characterization of the three reference standards demonstrated that all have a considerable number of antibodies that must be accounted for in an equimolar mix standard for the multiplex assay. The observed unitages of the MRS and other WHO standards were also verified at NIBSC, and an excellent agreement was observed. These unitages will provide opportunities to use these reference standards in multiplex assays. A second international standard has replaced the TE-3 reference standard, 13/240, which was characterized using a similar approach. The second international reference standard was also positive for antibodies against the other antigens. Nevertheless, the study provides a process and framework to establish a reference standard for MIAs.

Commercially available diagnostic kits for PT, FHA, PRN, DT, and TT are used widely to assess the antibody responses to aP-based combination vaccines. We also compared the multiplex assay results to commercially available ELISA kits, which are calibrated against ISs and provide unitages in IU/mL (5). A good concordance was observed among the multiplex and monoplex assay methods. However, the multiplex assay was more sensitive (2,000 times for PT, 1,000 times for FHA, 250 times for PRN, 330 times for DT, and 100 times for TT) for all the antigens than the commercially available ELISA kit.

Immunogenicity testing of aP-based combination vaccines is mainly based on detecting IgG antibody concentrations. Cell-based *in vitro* methods used for determining levels of toxin-neutralization antibodies for diphtheria and pertussis toxins have been reported in previous studies (30, 36, 37). These neutralization assays are based on determining the number of antibodies to PT and diphtheria antigens that inhibit the toxin-induced clustering of CHO and Vero cells, respectively (30). The CHO cell assay for pertussis toxin and Vero cell assay for diphtheria toxin is laborious, semi-quantitative, and less sensitive than ELISA-based readouts. Various studies have reported a positive correlation between the concentration of IgG antibodies and neutralization antibody titers (30, 36–38). We also studied the agreement between the IgG concentrations estimated by bead-based assay and toxin-neutralization antibodies for pertussis and diphtheria toxin antigens. The assay showed a positive correlation of > 0.75 with PT and DT neutralization assays. The correlation coefficient of 0.75 suggests that there was good agreement considering that both assays operate with different mechanisms and have different sensitivities and readouts, and this was consistent with findings in other studies (30). Immunogenicity testing of vaccines in clinics requires robust method development and validation. Existing regulatory guidance on bioanalytical method validation addresses vaccine immunogenicity assays in only a limited manner. The method was validated in accordance with FDA, EMA, and ICH M10 guidance (22–24). The pentaplex magnetic bead-based assay exhibited a wide, dynamic range and high sensitivity compared with commercially available assays. The assay showed excellent dilutional accuracy for all antigens, which is essential to understanding the full range of antibody responses to all five antigens in pre- and post-vaccinated samples. The validation also established the LOQs for all the antigens using international reference standards. In addition, the sample stability, robustness, and bead-to-bead lot consistency were also established during the validation. Among all the antigens, the PRN antigen was the most sensitive to assay conditions of plate hold time. However, the impact of plate hold time was minimal, as PRN was found to be stable for up to 12 h, which is considered sufficient to address any instrumental breakdowns during routine assay use. The assay was robust over different incubation temperatures and PE lots. This ensures that the assay is unaffected by minor variations, thereby ensuring that the performance of the assay is maintained on repeated use.

Overall, our study reports on a pentaplex assay validated for the simultaneous estimation of IgG antibody levels against PT, FHA, PRN, DT, and TT antigens in IU/mL using WHO reference standards. The assay exhibits a broader dynamic range (to allow quantification across all age groups) than commercially available diagnostic kits. The assay is quantitative with well-defined LOQ compared with the arbitrarily defined cut-offs in commercially available diagnostic kits. Our study also provides a characterization of WHO reference standards that can be used to determine the levels of antibodies present against all five antigens, allowing for their efficient use in multiplex assays. The increased sensitivity, reproducibility, and high throughput of this assay will enable the design of large and robust clinical studies for evaluating both natural and vaccine-induced immunity. Furthermore, since this assay was developed using Luminex technology, it provides opportunities for further expansion to include new antigens.

## Data availability statement

The original contributions presented in the study are included in the article/**Supplementary Material**. Further inquiries can be directed to the corresponding author.

## Ethics statement

The studies involving human participants were reviewed and approved by Independent Research Ethics Committee, Pune (IEC No. IRECP/015/2020. Written informed consent for participation was secured for this study in accordance with national legislation and institutional requirements.

## Author contributions

VR, LK, MG, PD, SB, SGu, SP, PP, HR, HS, US, SGa, KM, CA, AD-B, and LH participated in the conception, design, development, and validation of the assay, analysis, and interpretation of the data and drafting the article and revising it critically for important intellectual content. All authors contributed to the article and approved the submitted version.
