# Supplementary material for: Multiplexed bead-based assay for the simultaneous quantification of human serum IgG antibodies to tetanus, diphtheria, pertussis toxin, filamentous hemagglutinin, and pertactin
Source: Front Immunol. 2023 Jun 5;14:1190404. doi: 10.3389/fimmu.2023.1190404 (PMC10278353; doi:10.3389/fimmu.2023.1190404)
Supplement: Supplementary file 1 [file DataSheet_1.docx]

***Supplementary Material***

**Multiplexed Bead-Based Assay for Simultaneous Quantification of Human Serum IgG Antibodies to Tetanus, Diphtheria, Pertussis Toxin, Filamentous Haemagglutinin and Pertactin**

**Vishal Rathod^1^, Laxmikant Kadam^1^, Manish Gautam^1^, Prabhu Dasu Gumma^1^, Kevin Marke^2^, Cathy Asokanathan^2^, Alex Douglas-Bardsley^2^, Laura Hassell^2^, Sachin Bhandare^1^, Sumit Gupta^1^, Sameer Parekh^1^, Pramod Pujari^1^, Harish Rao^1,^ Hitt Sharma^1^, Umesh Shaligram^1^, Sunil Gairola^1*^**

^1^Clinical Bioanalytical Laboratory, Serum Institute of India Pvt. Ltd., Pune, Maharashtra, India

^2^Science, Research & Innovation, Medicines, and Healthcare Products Regulatory Agency, South Mimms, United Kingdom

***Correspondence**

Dr. Sunil Gairola

Clinical Bioanalytical Laboratory,

Serum Institute of India Pvt. Ltd.,

Hadapsar, Pune,

Maharashtra, India

E-mail: [sunil.gairola@seruminstitute.com](mailto:sunil.gairola@seruminstitute.com)

#
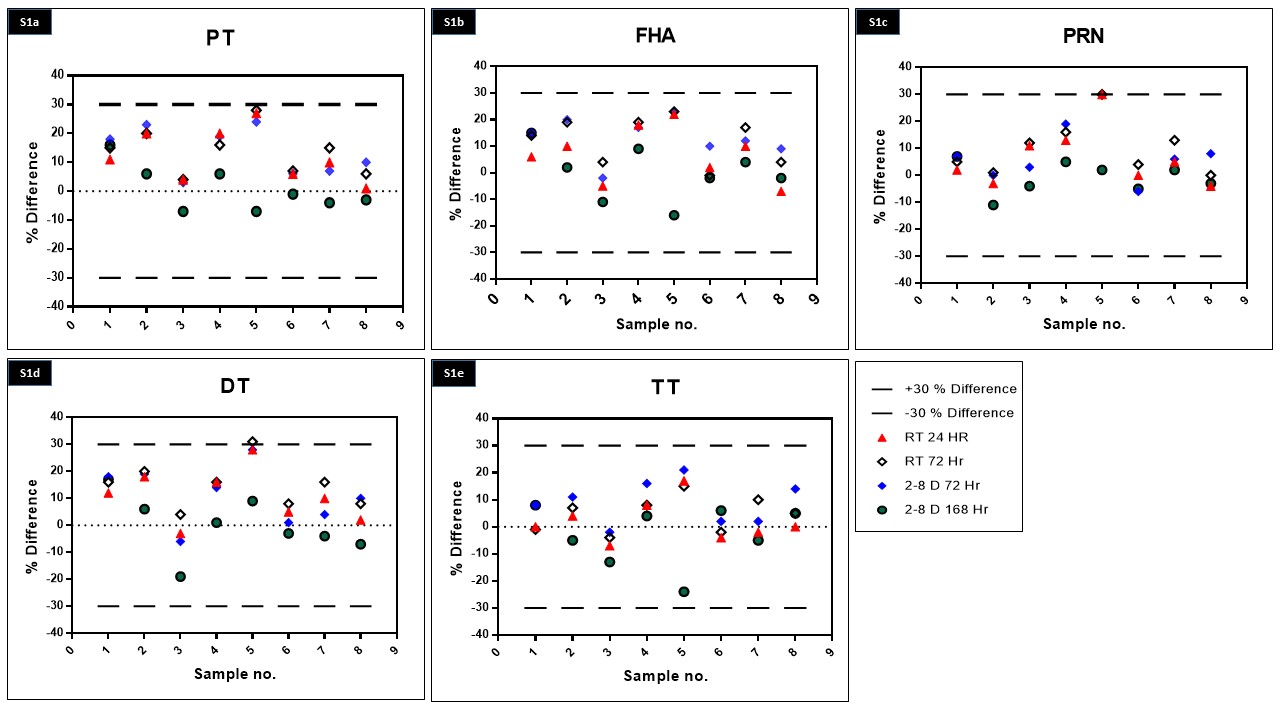


**Figure S1: Stability study at room temperature and 2-8ºC for PT, FHA, PRN, DT, and TT antigens**

The data on the X-axis represents the sample number, whereas the Y-axis represents the % difference. The concentrations observed are shown in different colors and shapes. The dotted line shows a ±30 % difference. DT, diphtheria toxoid, FHA, filamentous hemagglutinin; PRN, pertactin; PT, pertussis toxin; TT, tetanus toxoid

##
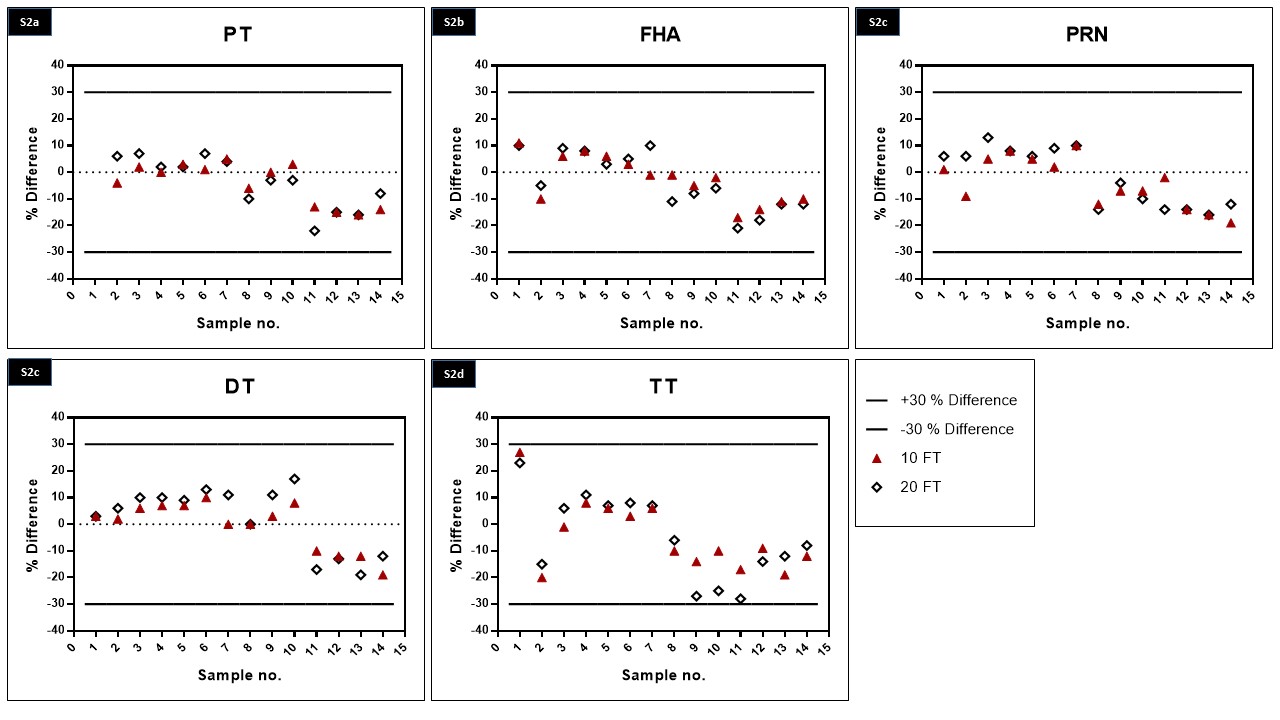
Figure S2: Freeze-thaw stability study with 10 cycles and 20 cycles at -20ºC for PT, FHA, PRN, DT, and TT antigens

The data on the X-axis represents the sample number, whereas the Y-axis represents the % difference. The concentrations observed are shown in different colors and shapes. Acceptance criteria of ±30 % difference was used to determine the impact of freeze-thaw cycles. DT, diphtheria toxoid; FHA, filamentous hemagglutinin; PRN, pertactin; PT, pertussis toxin; TT, tetanus toxoid


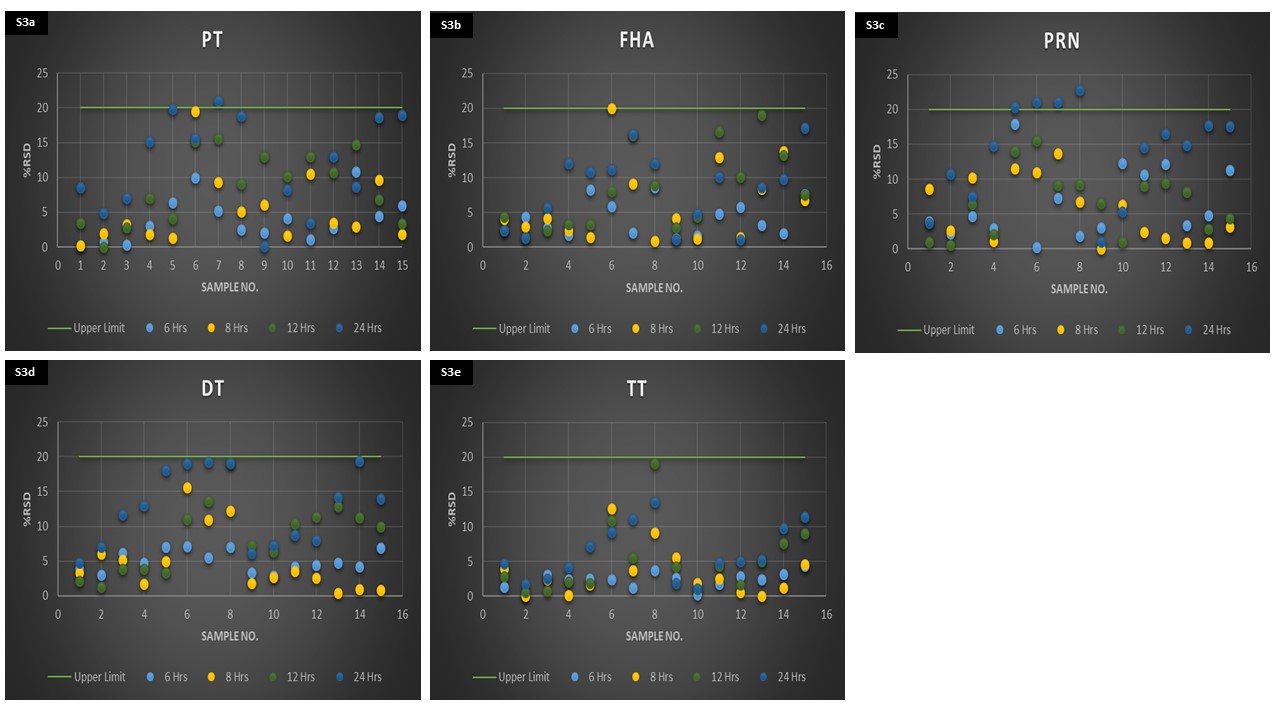


## Figure S3: Solution stability study

Solution stability was evaluated up to 24 hours for all the five antigens. X-axis represents the sample panel. Y-axis represents the % relative standard deviation (RSD) difference between assigned concentration and concentration obtained at different intervals. Among all the antigens, higher % RSDs were observed for PRN. DT, diphtheria toxoid; FHA, filamentous hemagglutinin; PRN, pertactin; PT, pertussis toxin; TT, tetanus toxoid
